# Development of a psychosocial-clinical nomogram to predict delayed medical help-seeking in women with stress urinary incontinence: a retrospective cohort study

**DOI:** 10.3389/fmed.2025.1691244

**Published:** 2025-11-12

**Authors:** Xuemei Luo, Yating Zhong, Dongmei Ai, Guiqiang Yin, Lu Mo

**Affiliations:** 1 Department of Urology, The Affiliated Hospital, Southwest Medical University, Luzhou, Sichuan, China; 2 Department of Oncology, The Affiliated Hospital, Southwest Medical University, Luzhou, Sichuan, China; 3 Operating Room, The Affiliated Hospital, Southwest Medical University, Luzhou, Sichuan, China

**Keywords:** stress urinary incontinence, delayed help-seeking, psychosocial factors, prediction model, nomogram

## Abstract

**Background:**

Delayed medical help-seeking is a common yet understudied behavior among women with stress urinary incontinence (SUI), potentially worsening symptom burden and diminishing quality of life. While clinical factors have been investigated, the contribution of psychosocial determinants remains poorly defined. This study aimed to identify key predictors and develop a validated nomogram for individualized risk estimation of delayed consultation.

**Methods:**

In this retrospective cohort study, we analyzed electronic health records of 1,400 adult women diagnosed with SUI at a tertiary medical center in Southwest China (2019–2023). Delayed help-seeking was defined as > 6 months between symptom onset and first medical consultation. Multivariable logistic regression, guided by backward stepwise selection and Akaike Information Criterion, was used to identify independent predictors. A nomogram was constructed and internally validated using 1,000 bootstrap iterations. Model performance was evaluated by AUC, calibration curves, and Hosmer–Lemeshow test.

**Results:**

Delayed help-seeking occurred in 58.0% of patients. Independent risk factors included older age (aOR = 1.03), higher BMI (aOR = 1.06), SUI duration > 12 months (aOR = 3.14), severe symptom severity (aOR = 2.06), and initial consultation with urology rather than gynecology (aOR = 1.94). Psychological predictors such as elevated anxiety scores (aOR = 1.06), perceived shame (aOR = 1.47), and social avoidance behavior (aOR = 1.66) were significantly associated with delay, while higher education was protective (aOR = 0.36). The nomogram showed strong discrimination (AUC = 0.855) and good calibration.

**Conclusion:**

Both clinical and psychosocial factors significantly influence delayed medical help-seeking in women with SUI. The proposed nomogram offers a validated, practical tool for early identification of high-risk individuals and may inform targeted interventions to reduce care delays and improve outcomes.

## Introduction

Stress urinary incontinence (SUI), characterized by involuntary urine leakage during activities that increase intra-abdominal pressure, is one of the most common pelvic floor disorders affecting women worldwide. The lifetime prevalence of stress urinary incontinence (SUI) in women varies widely across different populations, with estimates ranging from approximately 29 to 75%, depending on study design and case definitions (1). In a recent meta-analysis of Chinese women, the pooled prevalence of SUI was 24.5% (95% CI 22.5%–26.5%), underscoring the substantial burden in community settings (2). Despite its high burden, many affected women delay seeking medical care, which may exacerbate symptoms, diminish quality of life, and lead to progression of pelvic floor dysfunction (3, 4).

Timely medical consultation for SUI is influenced by a complex interplay of clinical severity, health beliefs, psychological distress, and social context. A growing body of literature highlights that women often perceive urinary incontinence as a normal part of aging, feel embarrassed, or fear stigmatization, all of which contribute to care-seeking delays (5–7). In community-based surveys conducted in China, only about 7.6% of women with mild-to-moderate SUI reported ever seeking medical help, with the most common reason for not seeking care being the belief that it was “normal” (8). Similarly, in a recent study from Ethiopia—a low-income country—approximately 73% of pregnant women experiencing urinary incontinence did not seek treatment, often attributing symptoms to normal pregnancy or lacking awareness of medical options (9).

Psychological factors such as anxiety, depression, perceived shame, and social withdrawal have emerged as key barriers to help-seeking, yet they are rarely quantified in predictive frameworks (10, 11). While some studies have identified individual predictors of delayed consultation using cross-sectional or qualitative designs (12, 13), few have developed validated models to estimate the likelihood of help-seeking delay. Notably, no existing prediction tool integrates both clinical and psychosocial determinants to stratify individual risk in women with SUI.

Nomogram-based prediction models have demonstrated strong utility in various urogynecologic contexts, including early postpartum stress urinary incontinence (SUI). For example, a nomogram developed to estimate the risk of early postpartum SUI after vaginal delivery achieved high discrimination (C-statistic 0.80; 95% CI: 0.74–0.85) and good calibration in both internal and validation cohorts (14). Likewise, a model combining pelvic floor ultrasound findings and clinical indicators yielded excellent AUC values—0.848 in the training set and 0.872 in validation—for postpartum SUI risk assessment (15). These tools offer interpretable, personalized risk estimates that may enhance patient counseling and clinical decision-making. Applying this methodology to help-seeking behavior in SUI represents an innovative extension with potential to guide early interventions for high-risk women.

Therefore, the present study aimed to (1) identify independent clinical and psychological predictors of delayed medical consultation among women with SUI, (2) construct a predictive nomogram based on these variables, and (3) internally validate the model’s discrimination and calibration using robust statistical techniques. By offering an individualized risk estimation tool, this study seeks to fill a critical gap in the early identification of patients who are likely to delay care and thus may benefit from targeted educational or psychological support.

## Materials and methods

### Study design and population

This retrospective cohort study was conducted at a tertiary academic medical center in Southwest China, in accordance with international reporting guidelines for observational studies (STROBE). We reviewed the electronic medical records of adult female patients who were diagnosed with stress urinary incontinence (SUI) between January 2019 and December 2023.

### Diagnostic criteria for stress urinary incontinence

To minimize subjectivity and ensure reproducibility, SUI was defined and confirmed according to contemporary clinical standards and recorded in the electronic medical record (EMR). The diagnosis required a combination of characteristic symptoms, physical examination findings, and physician confirmation. Specifically, patients reported involuntary urine leakage that occurred exclusively during increases in intra-abdominal pressure—such as coughing, sneezing, laughing, lifting, or exercising—with a frequency of at least once per week or an ICIQ-UI SF Q3 response of “once a week” or higher when available. During the pelvic examination, a positive cough stress test, defined as visible urine leakage from the urethral meatus while the bladder was comfortably filled (∼200–300 mL), served as supportive objective evidence. If the result was negative in the lithotomy position, the test was repeated in the standing position, and visible leakage in either posture was considered positive. The attending urologist or gynecologist subsequently documented a final clinical diagnosis of “stress urinary incontinence” in the EMR using standardized ICS terminology after integrating symptom history and pelvic examination findings. Urodynamic testing was not routinely required for uncomplicated cases but was performed at the clinician’s discretion in cases of diagnostic uncertainty (e.g., prior pelvic surgery, inconsistent symptoms and examination results, or suspected neurogenic disorders). When conducted, urodynamic stress incontinence (UDSI)—defined as urine leakage coincident with increased abdominal pressure in the absence of detrusor contraction—served as confirmatory evidence. All candidates underwent urinalysis to exclude urinary tract infection and a pelvic examination to rule out advanced pelvic organ prolapse (stage ≥ III, POP-Q) or urethrovaginal/vesicovaginal fistula; such cases were excluded during eligibility screening. To ensure data accuracy, two trained reviewers independently verified that all included cases met both the symptom and physician confirmation criteria, with discrepancies resolved by a senior clinician. Consistent with prevailing guidelines for uncomplicated SUI, our operational definition required stress-predominant symptoms and clinician confirmation, with a positive cough stress test as objective support when available.

### Inclusion and exclusion criteria

**FIGURE 1:**
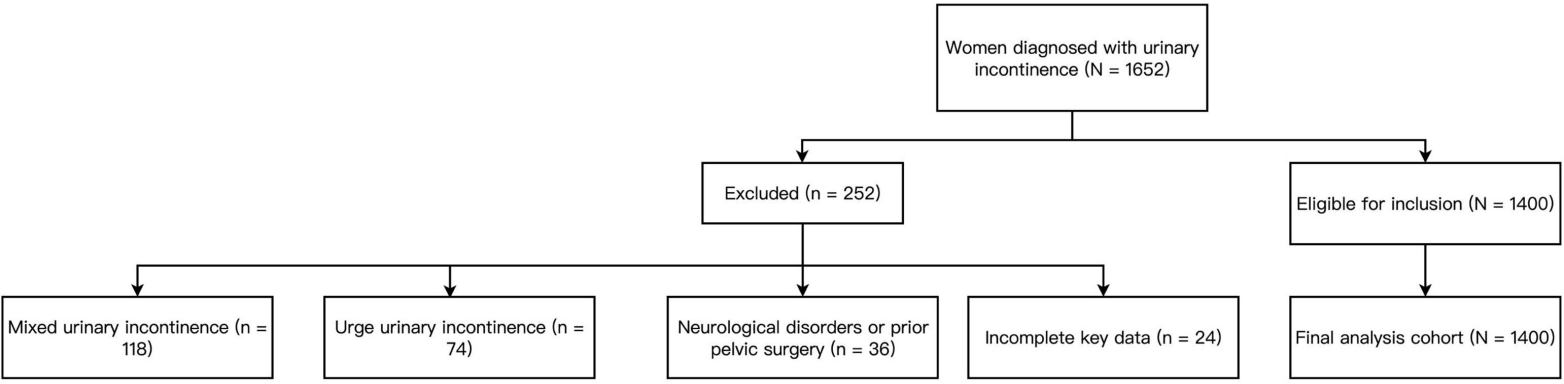
Flowchart of participant inclusion and exclusion for the final analytical cohort (*N* = 1,400). A total of 1,652 women diagnosed with urinary incontinence were screened for eligibility. After excluding 118 with mixed urinary incontinence, 74 with urge urinary incontinence, 36 with neurological disorders or prior pelvic surgery affecting bladder function, and 24 with incomplete data, 1,400 women with confirmed stress urinary incontinence were included in the final analysis.

Eligible participants were adult women (aged ≥ 18 years) with a primary diagnosis of stress urinary incontinence (SUI) and complete data on sociodemographic, clinical, and psychological variables. Women were excluded if they had mixed urinary incontinence (MUI) or urge urinary incontinence (UUI), neurological disorders or pelvic surgeries that could affect bladder function, or missing key exposure or outcome data. To ensure diagnostic specificity, women with MUI or UUI were excluded through a standardized two-step assessment. First, symptom-based screening was performed using the International Consultation on Incontinence Questionnaire–Urinary Incontinence Short Form (ICIQ-UI SF) and structured clinical interviews documented in the electronic medical record (EMR). Patients who reported urine leakage preceded or accompanied by a strong, sudden urge to void were classified as having urge-predominant or mixed incontinence and were excluded. Second, when urodynamic data were available, the presence of detrusor overactivity—defined as involuntary detrusor contractions during the bladder filling phase—served as an objective exclusion criterion for pure SUI. Women exhibiting both stress leakage and detrusor overactivity were categorized as MUI and excluded. In addition, patients with neurologic conditions (e.g., spinal cord injury, multiple sclerosis, Parkinson’s disease) or those with prior pelvic reconstructive surgery or radiation therapy were excluded to avoid secondary or neurogenic incontinence. All exclusions were confirmed by the attending physician during chart review, and two independent reviewers verified consistency during data abstraction. A total of 1,400 patients met the eligibility criteria and were included after rigorous chart review and data quality checks, following standardized steps of data extraction, cleaning, verification, and analysis by trained clinical researchers. The detailed inclusion and exclusion process is illustrated in Figure 1.

### Definition of delayed medical seeking

The primary outcome was delayed medical help-seeking, defined as a time interval of more than 6 months between the patient-reported onset of SUI symptoms and their first formal medical consultation. This threshold was based on previous literature and expert consensus, reflecting a clinically meaningful delay likely to impact disease progression and quality of life.

### Variables and measurement

Demographic data included age (continuous), BMI (kg/m^2^), education level (primary or below, secondary, college or above), employment status (employed vs. unemployed/retired), and marital status (married vs. unmarried/divorced/widowed). Clinical data included SUI symptom duration (months), dichotomized at > 12 months, SUI severity (mild/moderate/severe), prior conservative treatment (yes/no), and first consulted department (gynecology, urology, or other). SUI severity was determined by the attending physician based on both leakage frequency and pad usage, following criteria adapted from the International Consultation on Incontinence Questionnaire–Urinary Incontinence Short Form (ICIQ-UI SF): mild = occasional leakage (< 1 episode per day) or no pad use; moderate = daily leakage requiring 1–2 pads per day; severe = multiple episodes per day or ≥ 3 pads per day, or constant leakage interfering with daily activities. When available, these clinical impressions were cross-checked with ICIQ-UI SF total scores to ensure consistency (mild: 1–5; moderate: 6–12; severe: ≥ 13). Psychological measures included anxiety and depression scores using the validated Chinese versions of the Self-Rating Anxiety Scale (SAS) and Self-Rating Depression Scale (SDS). Perceived shame was measured on a 5-point Likert scale (1 = no shame, 5 = extreme shame), based on a culturally adapted instrument previously applied in pelvic floor research among Chinese women. Social avoidance and low self-esteem were evaluated during nurse-led structured interviews using a standardized psychosocial checklist developed through expert panel consensus. The checklist contained five behavioral items for social avoidance (e.g., avoiding public places, refraining from social gatherings, fear of odor or leakage in social contexts) and four for low self-esteem (e.g., feelings of inferiority, embarrassment about femininity, perceived loss of control, withdrawal from partner intimacy). Each item was scored dichotomously (yes/no). Presence of ≥ 2 affirmative responses within each domain was coded as “positive.” The instrument was pilot-tested in 60 women with urinary incontinence and demonstrated good inter-rater reliability (κ = 0.83) and internal consistency (Cronbach’s α = 0.81). All instruments demonstrated acceptable inter-rater reliability in the study sample (κ > 0.80) in a subsample quality audit.

### Data quality assurance

All data were independently extracted and cross-verified by two researchers. Discrepancies were resolved through consensus with a third reviewer. Less than 2% of data were missing across variables; thus, complete-case analysis was applied, as the low missingness was not expected to introduce bias.

### Statistical analysis

Descriptive statistics were used to summarize patient characteristics by delay status. Continuous variables were compared using Student’s *t*-test or Mann–Whitney U test, depending on normality. Categorical variables were analyzed using chi-square or Fisher’s exact test. Univariable logistic regression was conducted to assess the association between each variable and delayed medical seeking. Variables with *P* < 0.10 in univariable analysis were included in multivariable logistic regression using backward stepwise elimination based on the Akaike Information Criterion (AIC). All categorical predictors were dummy-coded with appropriate reference categories. Collinearity was checked using variance inflation factors (VIF), and none exceeded 2.5.

### Model development and validation

A predictive model was constructed based on the final multivariable regression results. The model’s discrimination performance was evaluated using the area under the receiver operating characteristic (ROC) curve (AUC), with 95% confidence intervals calculated via 1,000 bootstrap resamples. A nomogram was then developed based on the regression coefficients for individualized risk estimation. Model calibration was evaluated by plotting predicted versus observed probabilities across deciles of risk and assessing agreement using the Hosmer–Lemeshow test. Internal validation was conducted via bootstrap resampling with 1,000 iterations to estimate optimism-adjusted AUC and to assess model stability. All analyses were performed using R software (version 4.3.1), and a two-tailed *P* < 0.05 was considered statistically significant.

## Results

### Demographic, clinical, and psychological differences between groups

Table 1 presents the baseline demographic, clinical, and psychological characteristics of the 1,400 female patients with stress urinary incontinence (SUI), stratified by whether they sought medical help in a timely or delayed manner. Women in the delayed group (*n* = 812) were significantly older (54.3 ± 8.9 vs. 51.6 ± 8.0 years, *P* < 0.001), had higher BMI (25.1 ± 3.3 vs. 24.4 ± 3.0 kg/m^2^, *P* = 0.002), and were more likely to have lower educational attainment (*P* < 0.001). A higher proportion of delayed seekers were unemployed or retired (*P* = 0.016), and slightly more were unmarried, divorced, or widowed (*P* = 0.038). Clinically, delayed seekers had longer symptom duration (median 18 vs. 7 months, *P* < 0.001), more severe SUI (*P* < 0.001), and were less likely to have received conservative treatment before seeking care (*P* < 0.001). Psychologically, delayed seekers reported significantly higher anxiety (SAS), depression (SDS), and shame scores, and were more likely to exhibit social avoidance and self-reported low self-esteem (all *P* < 0.001) (Table 1).

**TABLE 1:** Demographic, clinical, and psychological characteristics of female patients with stress urinary incontinence (*N* = 1400).

Variable	Total (*N* = 1400)	Delayed seeking (*n* = 812)	Timely seeking (*n* = 588)	*P*-value
**Demographic characteristics**				
Age, mean ± SD (years)	53.2 ± 8.6	54.3 ± 8.9	51.6 ± 8.0	< 0.001
BMI, mean ± SD (kg/m^2^)	24.8 ± 3.2	25.1 ± 3.3	24.4 ± 3.0	0.002
Education level, n (%)	< 0.001			
– Primary or below	318 (22.7)	234 (28.8)	84 (14.3)	
– Secondary	726 (51.9)	440 (54.2)	286 (48.6)	
– College or above	356 (25.4)	138 (17.0)	218 (37.1)	
Employment status, n (%)	0.016			
– Employed	786 (56.1)	428 (52.7)	358 (60.9)	
– Unemployed/retired	614 (43.9)	384 (47.3)	230 (39.1)	
Marital status, n (%)	0.038			
– Married	1242 (88.7)	712 (87.7)	530 (90.1)	
– Unmarried/divorced/widowed	158 (11.3)	100 (12.3)	58 (9.9)	
**Clinical characteristics**				
Duration of SUI symptoms, median (IQR), months	13 (6–26)	18 (9–30)	7 (4–12)	< 0.001
SUI duration > 12 months, n (%)	834 (59.6)	612 (75.4)	222 (37.8)	< 0.001
SUI severity, n (%)	< 0.001			
– Mild	406 (29.0)	172 (21.2)	234 (39.8)	
– Moderate	652 (46.6)	388 (47.8)	264 (44.9)	
– Severe	342 (24.4)	252 (31.0)	90 (15.3)	
Previous conservative treatment, n (%)	312 (22.3)	122 (15.0)	190 (32.3)	< 0.001
First consulted department, n (%)	< 0.001			
– Gynecology	908 (64.9)	462 (56.9)	446 (75.9)	
– Urology	386 (27.6)	280 (34.5)	106 (18.0)	
– Other	106 (7.6)	70 (8.6)	36 (6.1)	
**Psychological measures**				
SAS score, mean ± SD	47.5 ± 9.2	50.2 ± 8.7	43.5 ± 8.9	< 0.001
SDS score, mean ± SD	48.1 ± 9.6	50.9 ± 9.0	44.1 ± 9.1	< 0.001
Perceived shame score, mean ± SD	3.2 ± 1.0	3.5 ± 0.9	2.8 ± 1.0	< 0.001
Social avoidance behavior, n (%)	578 (41.3)	412 (50.7)	166 (28.2)	< 0.001
Low self-esteem (self-reported), n (%)	390 (27.9)	288 (35.5)	102 (17.3)	< 0.001

SUI, stress urinary incontinence; SD, standard deviation; IQR, interquartile range; BMI, body mass index; SAS, Self-Rating Anxiety Scale; SDS, Self-Rating Depression Scale. “Delayed seeking” was defined as a time interval greater than 6 months between the onset of SUI symptoms and the first medical consultation. Perceived shame was measured using a 5-point Likert scale (1 = no shame, 5 = extreme shame). Social avoidance and self-esteem status were based on standardized patient interviews and nurse-reported clinical observations.

### Factors associated with delayed medical seeking: univariate analysis

As shown in Table 2, univariate logistic regression analysis identified multiple demographic, clinical, and psychological variables significantly associated with delayed medical help-seeking. Older age (OR = 1.04, 95% CI: 1.02–1.06, *P* < 0.001) and higher BMI (OR = 1.07, 95% CI: 1.02–1.12, *P* = 0.004) increased the odds of delay. Educational attainment was inversely associated with delay: patients with secondary education (OR = 0.63) or college and above (OR = 0.26) were less likely to delay (*P* < 0.001). Clinically, SUI duration > 12 months (OR = 4.86), severe SUI (OR = 3.81), and absence of prior conservative treatment (OR = 2.71) were strong predictors of delay. Consulting urology first (OR = 2.64) and reporting higher psychological distress, including anxiety (SAS), depression (SDS), shame, social avoidance, and low self-esteem, were all significantly associated with delayed medical care (all *P* < 0.001) (Table 2).

**TABLE 2:** Univariate logistic regression analysis of factors associated with delayed medical seeking in women with stress urinary incontinence (*N* = 1400).

Variable	Reference category	OR (95% CI)	*P*-value
**Demographic characteristics**			
Age (per 1-year increase)	–	1.04 (1.02–1.06)	< 0.001
BMI (per 1-unit increase)	–	1.07 (1.02–1.12)	0.004
Education level	Primary or below	–	< 0.001
– Secondary	0.63 (0.46–0.86)	0.004	
– College or above	0.26 (0.18–0.38)	< 0.001	
Employment status	Unemployed/retired	0.73 (0.58–0.91)	0.006
Marital status	Unmarried/divorced/widowed	0.78 (0.54–1.12)	0.174
**Clinical characteristics**			
SUI duration > 12 months	≤ 12 months	4.86 (3.91–6.06)	< 0.001
SUI severity	Mild	–	< 0.001
– Moderate	1.35 (1.01–1.80)	0.041	
– Severe	3.81 (2.70–5.37)	< 0.001	
Previous conservative treatment	Yes	2.71 (2.07–3.56)	< 0.001
First consulted department	Gynecology	–	< 0.001
– Urology	2.64 (2.00–3.47)	< 0.001	
– Other	1.52 (0.97–2.37)	0.068	
**Psychological measures**			
SAS score (per 1-point increase)	–	1.08 (1.06–1.10)	< 0.001
SDS score (per 1-point increase)	–	1.07 (1.05–1.09)	< 0.001
Perceived shame (per 1-point increase)	–	1.72 (1.53–1.93)	< 0.001
Social avoidance (Yes vs. No)	No	2.64 (2.09–3.34)	< 0.001
Low self-esteem (Yes vs. No)	No	2.62 (2.00–3.44)	< 0.001

OR, odds ratio; CI, confidence interval; BMI, body mass index; SUI, stress urinary incontinence; SAS, Self-Rating Anxiety Scale; SDS, Self-Rating Depression Scale. Education level and SUI severity were analyzed as categorical variables using dummy coding with the lowest category as reference. Continuous variables (age, BMI, SAS, SDS, shame) were treated linearly. Variables with *P* < 0.10 were considered for multivariable modeling in Table 3. “Delayed seeking” was defined as a time interval > 6 months between symptom onset and first consultation.

### Independent predictors of delayed medical help-seeking

In the multivariate logistic regression analysis (Table 3), several factors remained independently associated with delayed medical seeking. These included older age (adjusted OR = 1.03, *P* = 0.006), higher BMI (adjusted OR = 1.06, *P* = 0.030), SUI duration > 12 months (adjusted OR = 3.14, *P* < 0.001), severe SUI (adjusted OR = 2.06, *P* < 0.001), and consulting a urology department first (adjusted OR = 1.94, *P* < 0.001). Importantly, psychological variables such as higher SAS scores (adjusted OR = 1.06, *P* < 0.001), greater perceived shame (adjusted OR = 1.47, *P* < 0.001), and presence of social avoidance behavior (adjusted OR = 1.66, *P* = 0.002) also showed strong associations with delay. Higher education was protective: women with college or above education had significantly lower odds of delay (adjusted OR = 0.36, *P* < 0.001).

**TABLE 3:** Multivariate logistic regression analysis of factors independently associated with delayed medical seeking in women with stress urinary incontinence (*N* = 1400).

Variable	Adjusted OR (95% CI)	*P*-value
**Demographic characteristics**		
Age (per 1-year increase)	1.03 (1.01–1.05)	0.006
BMI (per 1-unit increase)	1.06 (1.01–1.12)	0.030
**Education level**		
– Secondary vs. primary or below	0.73 (0.51–1.05)	0.086
– College or above vs. primary or below	0.36 (0.24–0.56)	< 0.001
**Clinical characteristics**		
SUI duration > 12 months	3.14 (2.36–4.18)	< 0.001
**SUI severity**		
– Moderate vs. mild	1.22 (0.87–1.71)	0.245
– Severe vs. mild	2.06 (1.37–3.09)	< 0.001
Previous conservative treatment (No)	1.87 (1.36–2.57)	< 0.001
**First consulted department**		
– Urology vs. gynecology	1.94 (1.39–2.72)	< 0.001
– Other vs. gynecology	1.19 (0.70–2.01)	0.519
**Psychological measures**		
SAS score (per 1-point increase)	1.06 (1.03–1.08)	< 0.001
Perceived shame score (per point)	1.47 (1.26–1.71)	< 0.001
Social avoidance behavior (Yes vs. No)	1.66 (1.20–2.30)	0.002
Low self-esteem (Yes vs. No)	1.33 (0.96–1.85)	0.085

OR, odds ratio; CI, confidence interval; BMI, body mass index; SUI, stress urinary incontinence; SAS, Self-Rating Anxiety Scale. Education level and SUI severity were entered as dummy variables, with “primary or below” and “mild” as reference groups. The multivariable logistic regression model included all variables with *P* < 0.10 in univariate analysis (Table 2), and was built using backward stepwise selection based on the Akaike Information Criterion (AIC). Psychological and clinical variables were mutually adjusted to reduce confounding. Perceived shame was rated on a 5-point Likert scale. Delayed medical seeking was defined as a symptom duration > 6 months prior to first medical consultation. Variables with borderline statistical significance (0.05 < *P* < 0.10) were retained due to theoretical relevance and are marked in italics in the manuscript.

### Model performance and nomogram

**FIGURE 2:**
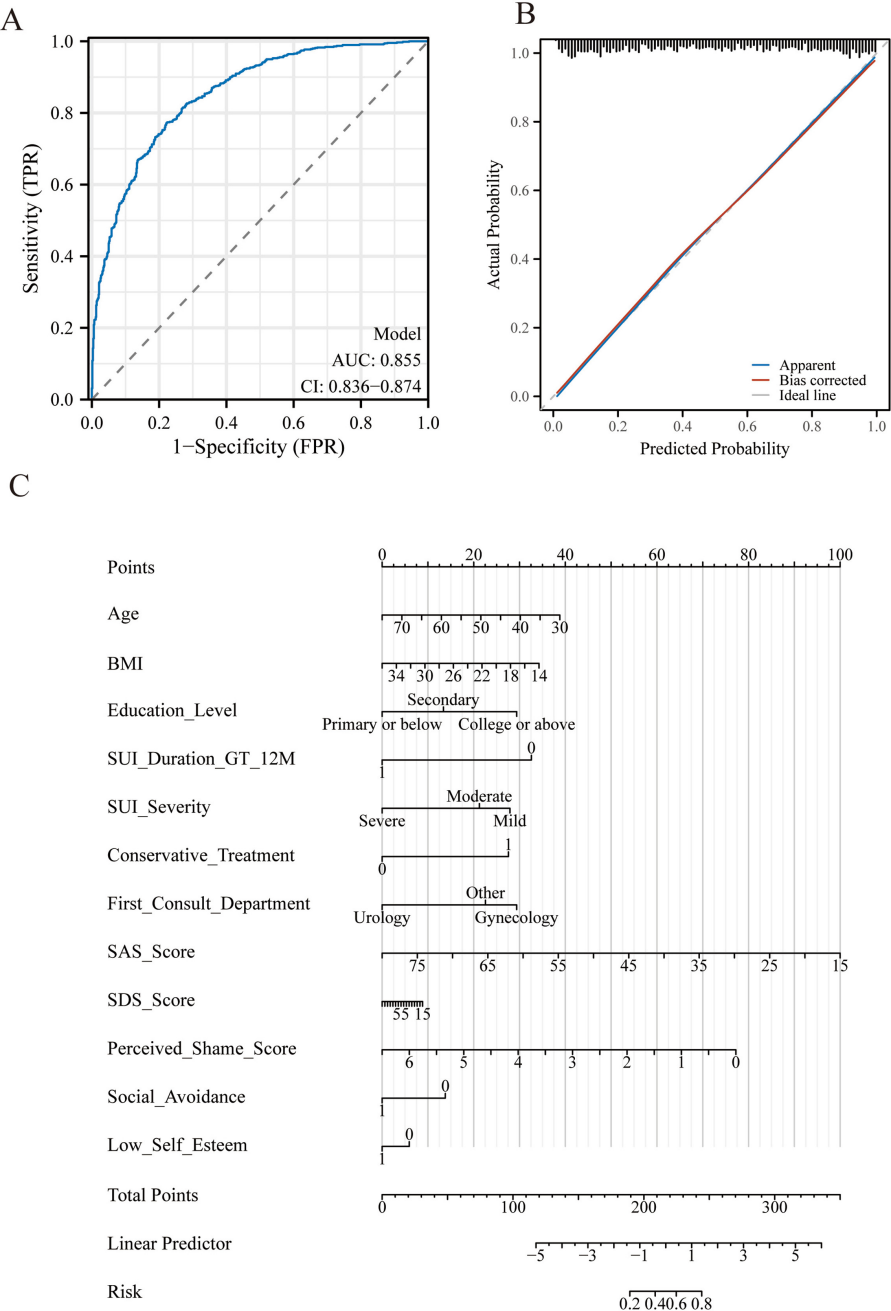
Model performance and predictive nomogram for delayed medical help-seeking in women with stress urinary incontinence (SUI). **(A)** Receiver operating characteristic (ROC) curve of the final multivariable logistic regression model, demonstrating good discrimination with an AUC of 0.855 (95% CI: 0.836–0.874). **(B)** Calibration plot of the model, showing close agreement between predicted probabilities and observed outcomes after 1,000 bootstrap resamples. The apparent curve (blue) and bias-corrected curve (red) are shown against the ideal 45° line (black). **(C)** Nomogram developed from the final model for individualized risk estimation. Each predictor is assigned a point value; the sum of points corresponds to the probability of delayed medical consultation, as indicated at the bottom risk scale.

The final predictive model demonstrated good discrimination, with an AUC of 0.855 (95% CI: 0.836–0.874) (Figure 2A). Calibration plots indicated excellent agreement between predicted and observed probabilities (Figure 2B). A nomogram was subsequently constructed to provide individualized risk estimates (Figure 2C), where higher total points correspond to a greater probability of delayed medical consultation. Each predictor’s regression coefficient from the final multivariable model was proportionally converted into a point scale, with the variable having the largest β value assigned 100 points as the reference. The total score for an individual patient was obtained by summing the points of all predictors, and the corresponding probability of delayed help-seeking was derived using the logistic transformation of the linear predictor.

## Discussion

This study presents a novel, integrative nomogram that quantifies the risk of delayed medical help-seeking in women with stress urinary incontinence (SUI) by incorporating both clinical and psychosocial variables. Our findings reveal that more than half of patients delayed seeking medical attention for over 6 months, and that delays are significantly influenced not only by symptom duration and severity but also by modifiable psychosocial constructs such as anxiety, perceived shame, and social withdrawal. These results provide a multidimensional understanding of care-seeking behavior in SUI and mark an important advancement in prediction modeling within urogynecology.

While previous studies have identified individual factors—such as embarrassment or symptom normalization—as barriers to seeking care, most have employed qualitative or cross-sectional designs (16, 17). Our study builds upon this foundation by quantitatively modeling these psychosocial variables alongside clinical data, producing a predictive tool with strong discrimination (AUC = 0.855) and calibration. Nomogram-based models in urogynecology have so far been applied almost exclusively to predict postpartum stress urinary incontinence (SUI) using obstetric factors and sonographic findings. For example, Xu et al. constructed a simple nomogram incorporating maternal age, parity, duration of second stage of labor, infant birthweight, and mode of delivery, achieving a C-statistic of 0.80 (95% CI: 0.74–0.85) in both development and validation cohorts (14). Similarly, Xiao et al. developed a combined clinical and transperineal ultrasound (TPUS) model—integrating BMI, delivery mode, episiotomy, pregnancy SUI, cystocele, and bladder-neck funneling—that demonstrated strong discrimination (AUC 0.848 in training and 0.872 in validation) (18). Our model captures a broader behavioral dimension, enabling more personalized risk stratification for clinical decision-making.

Several predictors identified in our model are well-established in literature, including age, higher BMI, symptom severity, and longer duration. These associations likely reflect physical and cognitive normalization of symptoms with aging and sociocultural barriers among older or less-educated individuals (19). Notably, women who initially consulted urology rather than gynecology were significantly more likely to delay seeking care, possibly reflecting symptom misattribution or fragmented care pathways. While recent studies have highlighted that non-linear or fragmented referral pathways—such as starting in urology without coordination with women’s health services—can increase the risk of delayed or suboptimal management of urinary incontinence (20), no previous work has explicitly quantified this effect in SUI populations, underscoring the novelty of our finding. It is also possible that this pattern reflects self-selection bias, wherein women who choose to consult urology first may differ systematically in demographic or psychosocial characteristics—such as age, symptom perception, or health-seeking preferences—from those who initially present to gynecology. This possibility warrants further exploration in future multicenter studies.

Crucially, our study demonstrates that psychosocial variables—especially shame and social avoidance—have independent predictive power beyond clinical severity. Although prior qualitative research has reported these factors as influential (21, 22), few quantitative models have incorporated them, despite strong theoretical rationale. For example, perceived shame may directly suppress disclosure or delay acknowledgment of symptoms, while social withdrawal may reduce interpersonal channels through which women receive support or encouragement to seek care. These psychosocial dimensions are particularly relevant in Chinese cultural contexts, where urinary incontinence is often considered a private and stigmatized issue, especially among older women (23). This cultural framing may explain the high proportion of delay (58%) observed in our cohort.

The use of validated instruments (SAS, SDS, and a culturally adapted shame scale) enhances the rigor of our psychosocial measures. Although these scales were originally developed for psychological screening, their predictive value in our logistic regression models justifies their inclusion as behavioral risk indicators in non-psychiatric populations. Nonetheless, future studies should explore whether non-linear modeling or threshold effects may better capture their influence. Moreover, while our nomogram was internally validated using bootstrap resampling, its generalizability remains to be tested in other geographic regions and cultural contexts. Psychosocial constructs such as shame and avoidance are inherently context-dependent, and their expression and salience may differ in Western societies, where greater openness about urinary disorders is more common (24).

This study has several limitations that should be considered when interpreting the findings. First, as a retrospective single-center analysis conducted at a tertiary hospital in Southwest China, the study’s findings have limited generalizability, representing a fundamental limitation of the design. The patient population may not fully reflect the broader demographic and cultural diversity of Chinese women, particularly those in rural regions or with different healthcare access patterns. The lack of multicenter data limits external validity and underscores the need for prospective validation across diverse geographic and sociocultural contexts. Second, although standardized abstraction protocols were used, recall bias remains a major concern because the definition of delayed help-seeking relied on the patient-reported interval between symptom onset and initial medical consultation. As urinary incontinence often develops insidiously, many women may not accurately recall the exact onset or progression of leakage, especially older patients or those with gradual symptom worsening. This could have led to misclassification of the outcome, with some cases of true delay being underestimated or overestimated. Despite these limitations, cross-checking symptom documentation and physician notes in the electronic medical record helped minimize but could not entirely eliminate recall bias. This could have affected the accuracy of delay classification, especially among older participants with longer disease courses. Furthermore, as this was an observational, retrospective study, the findings can only demonstrate associations rather than causal relationships. While the nomogram predicts the likelihood of delayed help-seeking, it cannot establish that psychological factors such as shame or anxiety directly cause the delay. Third, our dataset lacked important socioeconomic and behavioral variables—such as household income, health literacy, caregiving burden, and availability of social support—which may confound or mediate the association between psychological distress and help-seeking behaviors. The omission of these variables may have attenuated the model’s explanatory power and should be addressed in future studies using more comprehensive data collection frameworks. Fourth, while we applied internal validation via bootstrapping, the absence of external validation raises concerns regarding model overfitting and limits its current clinical applicability. Future research should aim to test the nomogram in independent cohorts with varied demographic and cultural profiles. Fifth, the structured checklist used to assess social avoidance and low self-esteem, although showing good internal consistency and inter-rater reliability, has not undergone external validation against standardized psychometric instruments, which may limit its construct validity. Moreover, psychosocial variables like perceived shame and social avoidance are inherently context-sensitive and may be interpreted differently across cultural backgrounds. As such, the model’s components may require cultural adaptation before being generalized to non-Chinese settings. Finally, despite using validated psychological instruments (SAS and SDS), we acknowledge the absence of structured psychiatric assessments, which may have introduced measurement error or misclassification in quantifying anxiety and depression levels.

Despite these limitations, our nomogram demonstrates promising clinical utility by enabling early identification of women at high risk for delaying medical consultation. Its integration into routine gynecological or primary care workflows could support pre-consultation triage and facilitate timely conversations about continence management. Embedding the model into electronic health record (EHR) systems, with automated risk alerts based on routinely collected demographic and psychological data, may allow clinicians to initiate targeted educational or behavioral interventions before the delay occurs. Importantly, such an approach aligns with global trends toward precision prevention in women’s health. Future prospective studies should assess the feasibility, acceptability, and cost-effectiveness of EHR-based risk stratification tools in real-world outpatient settings, as well as their impact on behavioral outcomes, care engagement, and pelvic floor morbidity.

In conclusion, this study provides strong evidence that both clinical severity and psychosocial burden contribute to delayed medical help-seeking among women with SUI. The proposed nomogram, which integrates anxiety, shame, and social avoidance into a validated predictive framework, offers a practical tool for early identification and targeted intervention. Its application in clinical and community settings may help shorten diagnostic delays and improve women’s quality of life. Future research should not only validate this model across diverse populations and cultural contexts but also develop intervention strategies to mitigate the identified barriers, thereby enhancing timely care-seeking and reducing the global burden of untreated pelvic floor disorders.

## Data Availability

The original contributions presented in this study are included in this article/supplementary material, further inquiries can be directed to the corresponding author.
